# Opposite response of blood vessels in the retina to 6° head-down tilt and long-duration microgravity

**DOI:** 10.1038/s41526-021-00165-5

**Published:** 2021-10-14

**Authors:** Giovanni Taibbi, Millennia Young, Ruchi J. Vyas, Matthew C. Murray, Shiyin Lim, Marina Predovic, Nicole M. Jacobs, Kayleigh N. Askin, Sara S. Mason, Susana B. Zanello, Gianmarco Vizzeri, Corey A. Theriot, Patricia Parsons-Wingerter

**Affiliations:** 1 Department of Ophthalmology and Visual Sciences, The University of Texas Medical Branch at Galveston, Galveston, TX USA; 2 NASA Johnson Space Center, Houston, TX USA; 3 Mori Associates, Ames Research Center, NASA, Moffett Field, Mountain View, CA USA; 4 Blue Marble Space Institute of Science, Space Biology Division, Space Technology Mission Directorate, Ames Research Center, NASA, Moffett Field, Mountain View, CA USA; 5 National Space Biomedical Research Institute, Ames Research Center, NASA, Moffett Field, Mountain View, CA USA; 6 Aegis Aerospace, Inc. Houston, Houston, TX USA; 7 KBR, NASA Johnson Space Center, Houston, TX USA; 8 Department of Preventive Medicine and Community Health, The University of Texas Medical Branch, Galveston, TX USA; 9 Low Gravity Exploration Technology, Research and Engineering Directorate, John Glenn Research Center, NASA, Cleveland, OH USA

**Keywords:** Preclinical research, Physiology

## Abstract

The Spaceflight Associated Neuro-ocular Syndrome (SANS), associated with the headward fluid shifts incurred in microgravity during long-duration missions, remains a high-priority health and performance risk for human space exploration. To help characterize the pathophysiology of SANS, NASA’s VESsel GENeration Analysis (VESGEN) software was used to map and quantify vascular adaptations in the retina before and after 70 days of bed rest at 6-degree Head-Down Tilt (HDT), a well-studied microgravity analog. Results were compared to the retinal vascular response of astronauts following 6-month missions to the International Space Station (ISS). By mixed effects modeling, the trends of vascular response were opposite. Vascular density decreased significantly in the 16 retinas of eight astronauts and in contrast, increased slightly in the ten retinas of five subjects after HDT (although with limited significance). The one astronaut retina diagnosed with SANS displayed the greatest vascular loss. Results suggest that microgravity is a major variable in the retinal mediation of fluid shifts that is not reproduced in this HDT bed rest model.

## Introduction

Recent NASA studies have established that serious risks for ocular/visual impairments are associated with microgravity exposure on the International Space Station (ISS), especially during long-duration missions^1–9^. Designated the Spaceflight Associated Neuro-ocular Syndrome (SANS, formerly Vision Impairment & Intracranial Pressure (VIIP) syndrome), ocular changes include optic disc edema (ODE), increased retinal and choroidal thicknesses, posterior globe flattening with hyperopic shift, choroidal flattening and folds, cotton wool spots, and decreased near visual acuity (hyperopic shift)^9^. Although the clinical incidence of SANS for these missions is approximately 15%, a subclinical incidence by ODE is estimated to occur in the majority of astronauts after missions of six months or longer^9^.

For this study, we tested the hypothesis that the headward fluid shifts associated with SANS and Head-Down-Tilt (HDT) bed rest, a well-established microgravity analog, are mediated at least in part by retinal blood vessels. The hypothesis further predicts that retinal vascular response occurs primarily in the more actively remodeling and fragile small vessels, as was observed previously for diabetic retinopathy and for microvascular response in other tissues to various pathological and physiological factors^10–25^. A well-established role of the microvasculature throughout the body is the regulation and maintenance of the intravascular and extravascular fluid balances within all tissues and organs, including the highly vascularized retina^26^. By this view, the microvascular endothelia and associated smooth muscle and pericytes are not only responders—but also active players and mediators—of fluid balances within a tissue.

Specifically for the study, vascular response in the retinas of healthy subjects undergoing 70 days of 6° HDT bed rest, an established ground-based spaceflight analog^27,28^, was compared to that of long-duration ISS crew members. The study protocol was essentially identical to that used previously for progression of diabetic retinopathy^19,25^. Arterial and venous patterns extracted from conventional ophthalmic images of the retina were mapped and quantified by several vascular parameters using the VESsel GENeration Analysis (VESGEN) software that was globally released by NASA in 2019^24,29^. The original VESGEN analysis was motivated by experimental observations in rodent and avian models that major molecular regulators such as VEGF and FGF induce unique ‘fingerprint’ or ‘signature’ vascular patterns within functionally diverse generations of fractally branching vascular trees^10–12,14–16,18,24^. The software quantification was therefore developed as a suite of complementary vascular parameters to characterize the fractal-based complexity of numerous uniquely branching vascular patterns. Parameters analyzed by VESGEN for the diabetic and current SANS retinal studies include the fractal dimension (*D*_f_), a sensitive estimator of vascular space-filling capacity, and vessel length density (*L*_v_) as a confirming measure^10–12,14,16,18,24,25,30^. To determine whether the small vessels were the primary responders, as was found for previous studies, the vascular results were further grouped by the software into branching generations of large (*G*_v1-4_) and small vessels of generations five or smaller (*G*_v≥5_). Results by VESGEN for the selective remodeling by small vessels in early diabetic retinopathy, the major blinding disease of working-aged adults, introduced new insights on disease progression that are encouraging for the development of early-stage regenerative therapies when progression may still be reversible^19^. A similar approach to early-stage monitoring by VESGEN of SANS progression could support countermeasure strategies for early-stage reversibility.

**Fig. 1:**
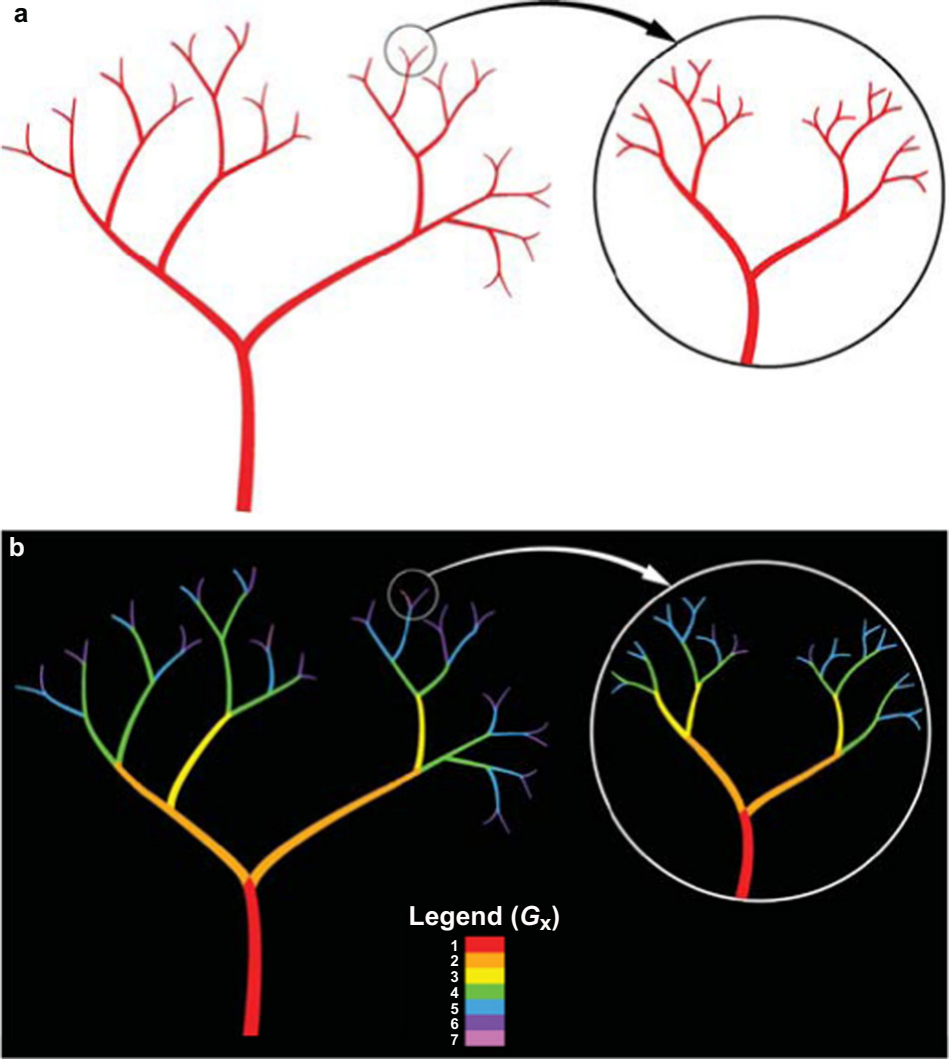
Self-similarity of fractally bifurcating patterns in branching vascular trees. **a** The fundamental pattern of self-similar bifurcational branching in vascular systems is illustrated by the schematic for successive ‘offspring’ generations (*G*_2_–*G*_7_) that branch from a single parent vessel (*G*_1_). The pattern displays iterative self-similarity at decreasing length scales^10,24,25,30–32,57,58^. The repeated pattern within the circle demonstrates how vessels can continue to branch below the level of image resolution. This phenomenon is a key feature of results reported here and elsewhere^24,25^ for both ISS crew members and HDT subjects, in which many small vessels were not detected due to limiting resolution of the Heidelberg Spectralis infrared (IR) ophthalmic imaging. **b** The VESGEN software assigned the vessels to seven generations of vascular branching and calculated the fractal dimension (*D*_f_) of the binary (red/white) pattern and its detail as 1.55 and 1.54, respectively (*D*_f_ of skeletonized images, 1.17 and 1.18). For a mathematically symmetric vascular fractal subject to the bifurcational branching rule $\mathop {\sum}\nolimits_{n = 0}^5 {} 2^n$ = 2^0^ + 2^1^ + 2^2^ + 2^3^ + 2^4^ + 25 = 63, the parent blood vessel would generate 32 small vessels in the sixth generation, *G*_6_ (*G*_1_–*G*_6_, 63 vessels total) for self-similar branching of decreasing vessel length. However, the vascular schematic depicts a more realistic biological fractal that is somewhat asymmetric. For this generalized example, iterative rules for vessel length and branching angle are not specified. In realistic human and vertebrate vascular branching, the bifurcational branching pattern is supplemented by the addition of many small ‘offshoot’ vessels to adequately vascularize the tissue (Fig. 2).

A fractal dimension is of fractional value, unlike the integers that specify the lines, planes, and solids of Euclidean geometry. Developed by B. Mandelbrot and others^31,32^, fractal mathematics describes complex space-filling patterns that include vascular and neuronal branching, coastlines, trees, and the species-specific vascular patterning of angiosperm leaves such as oak and maple^33^. Fractals often display the property of self similarity (Fig. 1), by which a characteristic pattern such as vascular bifurcational (dichotomous) branching is repeated at decreasing length scales. For a binary (black/white) vascular pattern in a 2D image, *D*_f_ is a fractional value lying between 1 and 2 that increases with vascular density. In previous human and other studies^10–12,14–16,18,24,30,34^, *D*_f_ ranged in skeletonized (linearized) vascular images from approximately 1.20–1.48, and often from 1.25–1.40. The skeletonized vascular representation, particularly of the more sparse vascular patterns used for this study, was found to be highly sensitive to physiological changes in vascular space-filling capacity^25^.

Reported in greater detail elsewhere^25^, the overall density of arteries and veins by *D*_f_ and *L*_v_ decreased significantly in the retinas of ISS crew members after six-month missions, especially that of small vessels. However, vascular decreases were highly variable among individual retinas. Decreases ranged from none or low up to 76% of the greatest loss in a retina diagnosed clinically with SANS by measures that include optic disc edema (ODE) and choroidal folds. A Subclinical Vascular Pathology Index (SVPI) was therefore established by comparing the vascular changes in the SANS retina to those of the other retinas. The results for ISS crew members are consistent with the highly variable clinical and subclinical incidence estimated for SANS by previously established measures such as ODE and peripapillary choroidal thickness^1–9^. The purpose of this report is to compare the retinal vascular response to 70 days of HDT with that of long-duration microgravity.

## Results

### Comparison of ISS and HDT vascular response

**Fig. 2:**
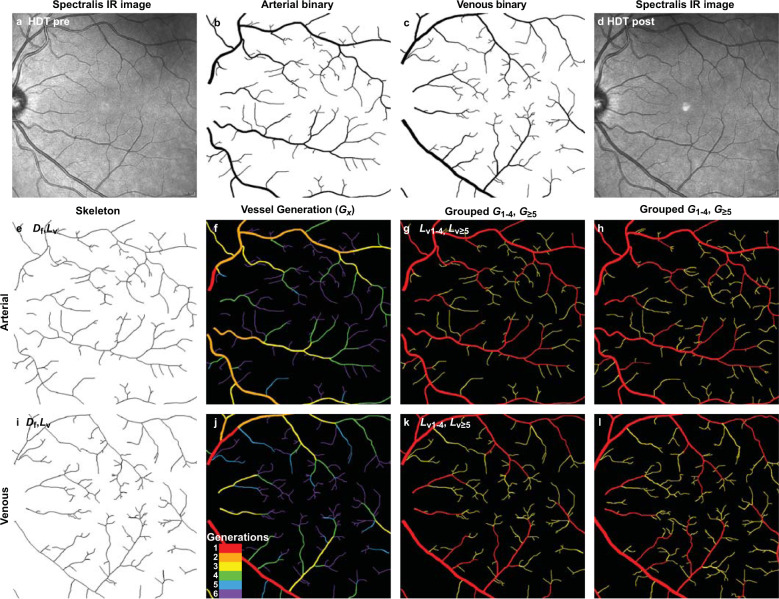
Mapping and quantification by VESGEN of retinal vascular patterning before and after HDT. **a** From the grayscale Spectralis IR image of the right retina of subject 2 acquired before 70 days of HDT (Supplementary Table 2), binary images of the **b** arterial and **c** venous trees were extracted to serve as the sole image inputs to VESGEN. **d** The same procedure was followed for the image acquired after 70 days of HDT. **e**, **i** The skeleton (centerline) of the pre-HDT arterial or venous tree was calculated by VESGEN that, together with the input binary image, generated additional maps of arterial and venous branching generations (**f**, **j**, legend). These generational maps were further grouped by the software into large (*L*_v1-4_, red) and small (*L*_v≥5_, yellow) vessels (**g**, **k**) for calculation of the final grouped vascular results (Table 2, Supplementary Table 2)^18,24^. The same analysis was followed for the post-HDT image (**d**, illustrated in **h** and **l**). Scale bars (**a**, **d**), 200 μm.

**Fig. 3:**
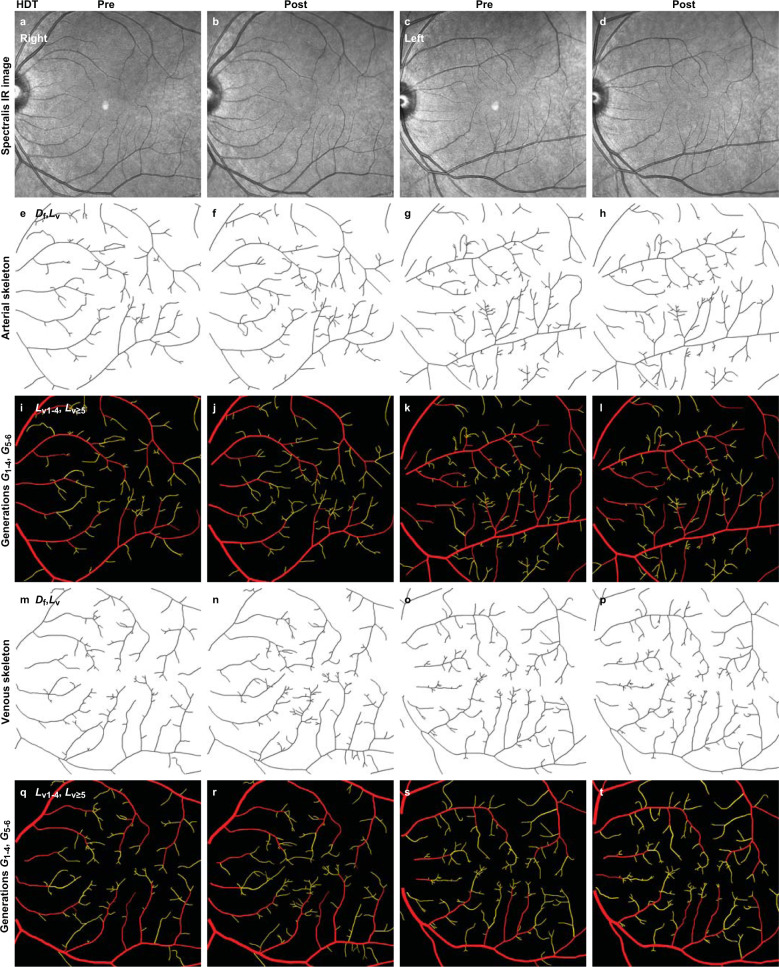
Representative increases in vascular density before and after 70 days of HDT. For the right and left retinas of subject 4 (Table 2; Supplementary Table 1), vascular density increased slightly according to mean values except for the left arterial density that remained essentially unchanged. As illustrated in Fig. 2, binary arterial and venous trees from grayscale retinal images (**a**–**d**) were analyzed by VESGEN as vascular skeletons (**e**–**h**, **m**–**p**) and branching trees grouped into large (*G*_v1-4_, red) and small (*G*_v5-6_, yellow) generations (**i**–**l**, **q**–**t**). Right arterial and venous length densities (*L*_v_) before HDT, 13.34 × 10^–4^ μm/μm^2^ and 13.37 × 10^–4^ μm/μm^2^; after, 13.97 × 10^–4^ μm/μm^2^ and 15.17 × 10^–4^ μm/μm^2^. Left arterial and venous length densities (*L*_v_) before HDT, 16.07 × 10^–4^ μm/μm^2^ and 15.07 × 10^–4^ μm/μm^2^; after, 16.00 × 10^–4^ μm/μm^2^ and 16.04 × 10^–4^ μm/μm^2^. Scale bars (**a**–**d**), 200 μm.

**Fig. 4:**
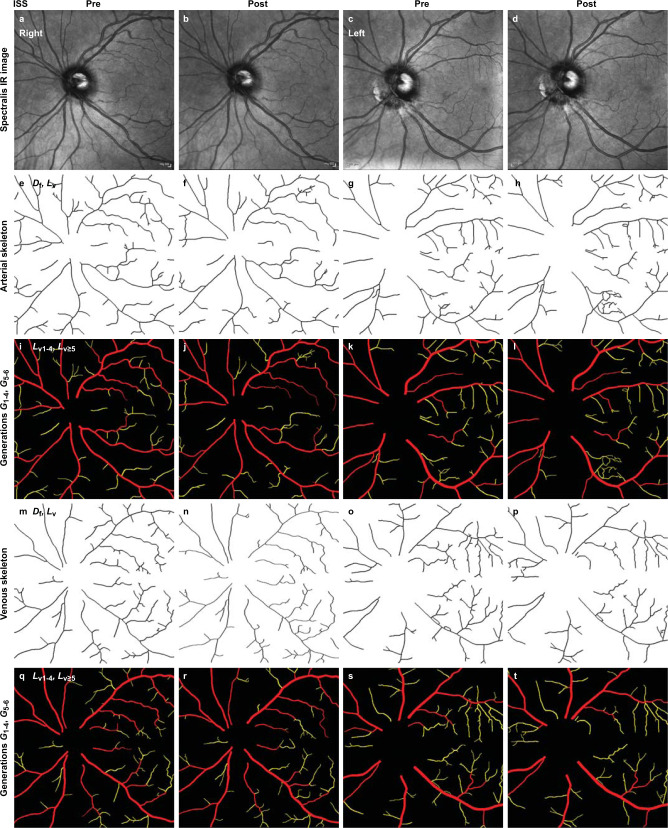
Representative vascular decreases in the retinas of an astronaut after 6 months in microgravity. Compared to a scale of 100% established by retinal arterio-venous loss in a crew member diagnosed with SANS^25^, arterial and venous density decreased in post-flight right and left retinas of this crew member by 35% and 8%, respectively. As for HDT subjects (Fig. 3), vascular trees extracted from grayscale images (**a**–**d**) were analyzed by VESGEN as skeletons (**e**–**h**, **m**–**p**) and branching trees grouped into large (*G*_v1-4_, red) and small (*G*_v5-6_, yellow) generations (**i**–**l**, **q**–**t**). Pre- to post-flight arterial and venous results for *D*_f_ in right retina, 1.38 to 1.35 and 1.36 to 1.35, respectively; for *L*_v_, 14.4 to 12.6 × 10^–4^ μm/μm^2^ and 13.0 to 12.4 × 10^–4^ μm/μm^2^. Similarly for left retina, 1.35 to 1.35 and 1.34 to 1.33; 12.4 to 12.6 × 10^–^^4^ μm/μm^2^ and 11.6 to 11.1 × 10^–4^ μm/μm^2^. For astronauts, Spectralis IR imaging was centered on the optic disc, not on the macula as for HDT subjects. Scale bars (**a**–**d**), 200 μm.

Changes in the arterial and venous patterning of ten retinas in five healthy subjects before and after 70 days of HDT bed rest at NASA’s Flight Analogs Research Unit (FARU)^27^ and of 16 retinas in eight U.S. crew members before and after 6-month missions to the ISS were mapped and quantified. The 35° Spectralis IR imaging conditions for the HDT and ISS cohorts were highly similar, and include performing the eye exam and imaging in the standard seated position post-HDT and post-ISS (Supplementary Table 1). The comparison of vascular patterns within images focused on the macular region in the HDT and on the optic disc in astronauts was not ideal but was limited by the availability of relevant image sets. Nonetheless, the macular region is almost completely captured by the field of view (FOV) in both image sets. Importantly, given the relatively low level of Spectralis IR resolution, the lack of vessels in the foveal avascular zone appears similar in both the HDT and ISS FOV (Figs. 2–4), where the smallest vessels are not captured by either imaging. Overall, comparison of the HDT and ISS vascular image sets was judged to be reasonable because magnification and imaging modality are essentially equivalent. In addition, the image sets were acquired in follow-up mode, resulting in alignment across test sessions within individuals.

The opposite patterns of vascular response by *D*_f_, *L*_v_, and *L*_v≥5_ in the HDT and astronaut retinas were compared by mixed models (Table 1). While crewmembers experienced pre- to post-mission vascular decreases, HDT subjects displayed slight increases in the same parameters before and after bedrest. As hypothesized, these differences in vascular response resulted primarily from changes in the small vessels as measured by vessel length density (*L*_v≥5_). The pre-to-post density of larger arteries and veins (*L*_v1-4_) did not differ meaningfully between the HDT and ISS cohorts.

**Table 1:** Comparison of change in arterial and venous density in retinas of 6° HDT subjects and ISS crew members.

Vascular tree	Parameter	Symbol	F-test time*group df 3 and 37[value (*p*-value)]^a^	Crew pre to post change, mean ± SE	HDT pre to post change, mean ± SE	*P* value for comparison of changes
Arterial	Fractal dimension	*D*_f_	3.50 (0.0248)	−0.016 ± 0.007^b^	+0.017±0.008	0.0027
	Length density *All vessels*	*L*_v_	10.32 (<0.0001)	−8.2E-5 ± 3.7E-5^b^	+5E-5 ± 3.9E-5	0.0215
	*Large vessels*	*L*_v1–4_	0.22 (0.8790)	+1E-5 ± 1.8E-5^b^	+7.73E-6 ± 1.8E-5	0.9056
	*Small vessels*	*L*_v≥5_	15.46 (<0.0001)	−9.2E-5 ± 3E-5^b^	+4E-5 ± 2.5E-4	0.0020
Venous	Fractal dimension	*D*_f_	3.85 (0.0170)	−0.015 ± 0.005^b^	+0.017 ± 0.009	0.0035
	Length density *All vessels*	*L*_v_	17.83 (<0.0001)	−6.5E-5 ± 2.9E-5^b^	+6E-5 ± 2.9E-5	0.0107
	*Large vessels*	*L*_v1–4_	2.09 (0.1181)	+1E-5 ± 9.3E-6^b^	+2E-5 ± 1.5E-5	0.5555
	*Small vessels*	*L*_v≥5_	11.72 (0.0001)	−7.5E-5 ± 3.1E-5^b^	+4E-5 ± 4E-5	0.0252

^a^Differences in vascular parameters generated by VESGEN for 10 retinas of five subjects before and after 70 days of HDT bed rest compared to 16 retinas of eight ISS crew members before and after 6-month missions to the ISS were estimated using expected marginal means of mixed-effects models, where df denotes degrees of freedom for F distributions.

^b^Results reproduced from study of retinal vascular loss in ISS crew members with and without SANS^25^ *D*_f_, fractal dimension (dimensionless), *L*_v_ overall vessel length density, *L*_v1-4_ vessel length density of generations one to four, *L*_v≥5_ vessel length density of generations ≥5. By study hypothesis, vascular response was predicted primarily for small (*L*_v≥5_) but not large (*L*_v__1-4_) vessels.

### Detailed analysis of HDT vascular response

**Fig. 5:**
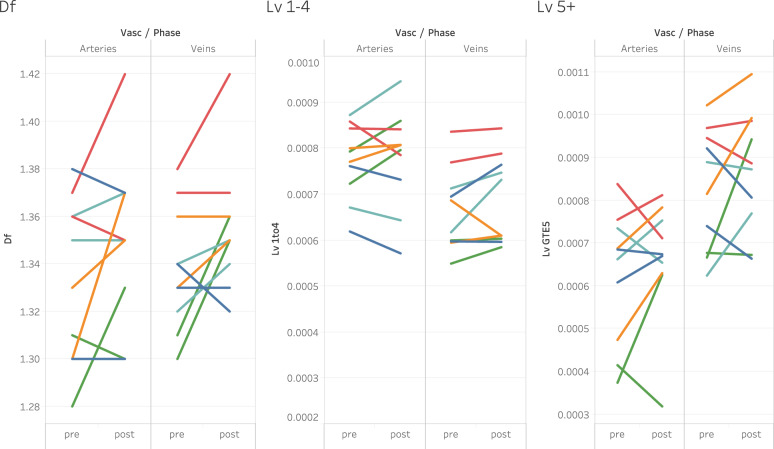
Variability in vascular response of individual HDT subjects after 70 days of bed rest. Comparison of values of vascular parameters *D*_f_, *L*_v1-4_, and *L*_v>5_ for each of the 10 de-identified retinas, and their pre-post change, before and after HDT. The two retinas of an individual subject are denoted by the same color. As hypothesized, the larger vessels (*L*_v1-4_) did not remodel as greatly as the smaller vessels (*L*_v>5_). The variability of retinal vascular response is consistent with the variability measured for other ocular changes in both astronaut and HDT populations such as ODE and retinal and choroidal thickness. In the *L*_v1-4_ column for veins, lower blue and orange bars overlap with green bar.

Trends of increased density in post-HDT retinal vessels detected by the sensitive VESGEN analysis were modest (Table 2; individual fractal results in Supplementary Table 2). Arterio-venous densities increased in 50% of the HDT retinas by a cutoff value of Δ*D*_f_ > |0.02 | (Table 2). This value was derived from previous studies^10–12,14,16,18,24,25,30^ as indicative of significant change in vascular space-filling capacity. By the Δ*D*_f_ cutoff, no vascular decreases were found. Transitional increases in *D*_f_ were measured for two retinas and a transitional decrease, for only one retina. Results for HDT are illustrated by modest increases in retinal arterial and venous densities of three retinas that are consistent with mean changes in the population (Figs. 2–3; Supplementary Table 2). Overall arterial and venous length densities (*L*_v_) in Fig. 2, for example, were quantified as 14.88 × 10^–4^ μm/μm^2^ and 14.10 × 10^–4^ μm/μm^2^ before HDT, compared to 15.91 × 10^–4^ μm/μm^2^ and 16.03 × 10^–4^ μm/μm^2^ after HDT. Varying values in *D*_f_, *L*_v1-4_, and *L*_v>5_ before and after HDT for each of the 10 de-identified retinas are further illustrated (Fig. 5). No significant changes in vessel diameter and tortuosity were measured for the HDT and ISS retinas, although subtle changes in these vascular characteristics may not have been captured by the relatively low Spectralis IR imaging.

**Table 2:** Change of arterial and venous density in retinas of 6° HDT subjects after 70 days of bed rest.

Vascular tree	Symbol	HDT time	Results, Mean ± SE	Pre-to-post change, Mean ± SE	*P* value
Arterial	*D*_f_	Pre Post	1.334 ± 0.012 1.351 ± 0.011	+0.017 ± 0.008	0.031
	*L*_v_	Pre Post	1.39E-3 ± 7.0E-5 1.44E-3 ± 4.9E-5	+5E-5 ± 3.9E-5	0.233
	*L*_v1–4_	Pre Post	7.71E-4 ± 2.3E-5 7.79E-4 ± 2.9E-5	+7.76E-6 ± 1.8E-5	0.677
	*L*_v≥5_	Pre Post	6.24E-4 ± 6E-5 6.63E-4 ± 4.5E-5	+4E-5 ± 2.5E-5	0.122
Venous	*D*_f_	Pre Post	1.338 ± 0.011 1.355 ± 0.010	+0.017 ± 0.009	0.058
	*L*_v_	Pre Post	1.49E-3 ± 7.5E-5 1.56E-3 ± 6.0E-5	+6.0 ± 3.8E-5	0.100
	*L*_v1–4_	Pre Postt	6.66E-3 ± 3.4E-5 6.88E-3 ± 3.7E-5	+2E-5 ± 1.5E-5	0.153
	*L*_v≥5_	Pre Pos	8.27E-4 ± 4.7E-5 8.69E-4 ± 4.1E-5	+4E-5 ± 4E-5	0.230

HDT-related changes were estimated for HDT subjects (*n* = 5, 10 retinas) using expected marginal means from mixed models. *D*_f_, fractal dimension (dimensionless), *L*_v_ overall vessel length density, *L*_v1-4_ vessel length density of generations one to four, *L*_v≥5_ vessel length density of generations ≥5. By study hypothesis, vascular response was predicted primarily for small (*L*_v≥5_) but not large (*L*_v1-4_).

### Establishing Subclinical Vascular Pathology Index (SVPI) from SANS incidence

Importantly as detailed elsewhere^25^, the retina of one crew member displaying the largest vascular decreases was also the only retina diagnosed post-flight with SANS by established clinical measures such as ODE and choroidal folds. Globe flattening, ODE and increases in refractive error total retinal thickness (TRT) were greatest in this SANS retina. No changes in visual acuity were reported by any crew member^25^. No statistically significant association between intraocular pressure (IOP) and VESGEN parameters were found. From this retina, the SVPI was established to quantify relative decreases in vascular density of the other 15 retinas. By the SVPI, three other retinas displayed significant vascular decreases from 67 to 76% of vascular loss in the SANS retina^25^. To illustrate the mean values of modest vascular decrease in most of the ISS cohort (Fig. 4)^25^, pre- to post-flight arterial and venous changes by *D*_f_ in the right retina of this crew member were 1.38 to 1.35 and 1.36 to 1.35, respectively; by *L*_v_, 44.55 to 39.11 × 10^–4^ μm/μm^2^ and 40.13 to 38.34 × 10^–4^ μm/μm^2^. For the left retina, pre- to post-flight arterial and venous values for *D*_f_ were 1.35 to 1.35 (no change) and 1.34 to 1.33; for *L*_v_, 38.29 to 39.00 × 10^–4^ μm/μm^2^ and 36.69 to 34.50 × 10^–4^ μm/μm^2^. For a recent publication by Laurie et al.^5^, the earliest signs of optic disc edema were determined to be a change in TRT (0–250 µm) greater than 19.7 µm. Based on this definition of optic disc edema currently accepted by NASA^7^, 60% of the crew members in the current study displayed one or more signs of SANS (3/5 crew members; TRT data were not available for 3 crew members).

### Association between VESGEN results for HDT with other ophthalmic measures

Mixed-effects models were used to evaluate the association between the VESGEN vascular results and ophthalmic measures in the HDT cohort that include LogMAR visual acuity, spherical equivalent, IOP, Spectralis OCT retinal nerve fiber layer thickness, and peripapillary and macular total retinal thicknesses (Table 3). Given the small sample size (*n* = 5, 10 retinas) and large number of ophthalmic measures of interest, this evaluation can only be considered an exploratory analysis to identify possible associations for further study. Unadjusted *p*-values are reported alongside false discovery rates (FDR) to put the multiple testing conducted around these measures in context. Small, subclinical increases in peripapillary retinal thickness were reported previously after 70-day HDT bed rest^27^.

**Table 3:** Association of ocular measures and arterial and venous density in retinas of 6° HDT subjects after 70 days of bed rest.

Symbol	Ocular measure	Arterial			Venous		
		Results, Coeff ± SE	*P* value	FDR	Results, Coeff ± SE	*P* value	FDR
*D*_f_	RNFL thickness	−9E-5 ± 6.8E-4	0.90	0.97	−2.3E-4 ± 1.0E-3	0.83	0.91
	Peripapillary thickness	1.0E-3 ± 2.9E-4	0.01	0.06	2.7E-6 ± 3.4E-4	0.99	0.99
	Macular thickness	1.1E-3 ± 1.5E-3	0.49	0.74	3.5E-4 ± 5.5E-4	0.55	0.74
	IOP	6.2E-4 ± 1.8E-3	0.74	0.84	3.5E-3 ± 1.1E-3	0.02	0.09
	Spherical equivalent refraction	−1.1E-2 ± 1.9E-2	0.58	0.74	9.7E-3 ± 3.3E-3	0.02	0.09
	Near LogMAR	0.186 ± 0.155	0.26	0.55	0.070 ± 0.090	0.48	0.74
	Distance LogMAR	0.397 ± 0.072	0.00	0.02	0.107 ± 0.028	0.00	0.06
*L*_v1–4_	RNFL thickness	3.6E-6 ± 2.0E-6	0.11	0.32	2.7E-6 ± 2.0E-6	0.21	0.52
	Peripapillary thickness	1.2E-6 ± 1.2E-6	0.34	0.65	9.6E-7 ± 1.3E-6	0.48	0.74
	Macular thickness	2.1E-6 ± 1.6E-6	0.25	0.54	1.3E-6 ± 2.1E-6	0.55	0.74
	IOP	−2.1E-6 ± 3.5E-6	0.57	0.74	−2.1E-6 ± 4.8E-6	0.67	0.80
	Spherical equivalent refraction	−1.6E-6 ± 1.7E-5	0.93	0.97	−2E-5 ± 3.1E-5	0.54	0.74
	Near LogMAR	3.3E-4 ± 1.1E-4	0.02	0.09	−6E-5 ± 1.3E-4	0.65	0.80
	Distance LogMAR	1.9E-4 ± 1.7E-4	0.32	0.64	−3.5E-4 ± 2.3E-4	0.17	0.44
*L*_v≥5_	RNFL thickness	8.8E-6 ± 4.1E-6	0.51	0.74	5.3E-6 ± 2.9E-6	0.10	0.32
	Peripapillary thickness	3.0E-6 ± 0	0.02	0.09	3.0E-6 ± 0	0.00	0.06
	Macular thickness	2.8E-6 ± 4.8E-6	0.58	0.74	2.0E-6 ± 5.5E-6	0.73	0.84
	IOP	4.9E-6 ± 6.7E-6	0.49	0.74	1.6E-5 ± 1.2E-5	0.23	0.53
	Spherical equivalent refraction	−2.8E-6 ± 1.0E-4	0.98	0.99	9.9E-5 ± 5.2E-5	0.09	0.32
	Near LogMAR	−1.3E-3 ± 4.3E-4	0.02	0.09	−8.4E-4 ± 4.8E-4	0.12	0.34
	Distance LogMAR	1.2E-3 ± 5.9E-4	0.08	0.30	9.4E-4 ± 2.7E-4	0.01	0.06

Comparison of ocular measures and vascular parameters generated by VESGEN for 10 retinas of five subjects who underwent 70 days of HDT bed rest. *D*_f_ fractal dimension (dimensionless). *L*_v_ overall vessel length density, *L*_v1-4_ vessel length density of generations one to four, *L*_v≥5_ vessel length density of generations ≥5. FDR false discovery rate.

## Discussion

Trends for microvascular response within the retina were found by VESGEN to be opposite for 70 days of 6° HDT bed rest and six months of microgravity on the ISS. By several confirming measures of small and overall vessel density, vascular patterning increased slightly with HDT and decreased significantly in astronauts. Subtle vascular changes or differences that are not apparent by visual inspection of the clinical grayscale images were quantified. Retinal vascular adaptations in the two healthy, relatively young populations were generally modest, compared to much greater vascular changes in potentially blinding vascular-dependent pathologies such as diabetic retinopathy^19^ or age-related macular degeneration^35^ that typically develop over years.

Results for subtle vascular change by the sensitive VESGEN analysis provide preliminary confirmation of our hypothesis that the retinal microvasculature necessarily remodels to accommodate the headward fluid shifts incurred during microgravity and HDT and furthermore, that vascular adaptations are mediated primarily by the more fragile, actively remodeling smaller vessels^18,19,24–26^. Potential mechanisms for differences in vascular response include: (1) expansion (dilation) of small vessel diameters above the limits of image resolution in HDT and contraction below these limits in microgravity, (2) other processes of vascular remodeling such as vaso-obliteration (microvascular rarefaction), or (3) a combination of both. Given the high metabolic cost of vessel loss, neovascularization and angiogenesis, as well as the healthy status of the subjects, the more likely explanation may be that the vessel diameters simply decreased in microgravity and expanded during HDT. Veins typically are of wider caliber than arteries. Overall, venous densities changed less than arterial densities under both HDT bed rest and ISS mission conditions (Tables 1 and 2)^25^. At the limits of image resolution, perhaps the smaller arterial diameters resulted in increased detection of change, both positive and negative, compared to venous diameters.

In both the HDT bed rest and ISS crew member populations, the retinal vascular response was variable among subjects as well as among the left and right eyes of a single individual. This result conforms with the incidence of SANS as unilateral, bilateral, and of marked variability among astronauts, when diagnosed by clinically established measures such as optic disc edema, cotton wool spots, globe flattening, and total retinal and choroidal thicknesses^1,9^. The results also conform with current ophthalmic findings in astronauts that manifest as a spectrum of cerebral responses and vision impairment^1,9^. For example, although cerebral venous outflow in the internal jugular vein has been shown to stall and even become retrograde during spaceflight, this result is not uniform for all crew members^6,36^. By OCT, subclinical increase in peripapillary retinal thickness as an indicator of ODE also varied among the HDT subjects of this study^27^. Nonetheless, significant differences in peripapillary retinal thickness were measured after the 14 and 70-day FARU HDT campaigns, together with increased superior, nasal, and inferior peripapillary retinal thicknesses after the 70-day HDT compared to 14 days of HDT^5^.

Recently, NASA modified the bed rest analog protocol for supine 6° HDT used for the FARU study that is analyzed here. By the newer protocol, subjects maintain a strict HDT posture to sustain the cephalad pressure gradient (i.e., unlike for FARU, subjects retain 6° HDT even while consuming meals). The OCT analysis of a recent 30-day 6° HDT study at envihab^5^ was conducted under the stricter posture control that also included mild hypercapnia (0.5% CO_2_ above ambient) to further mimic the ISS environment that contains elevated levels of CO_2_. Increases in ODE indicated by increased peripapillary thickness after HDT were greater than in crew members by OCT measurements aboard the ISS, as were trends for greater increases in total choroidal thickness in crew than HDT. In addition, increases in TRT for the strict hypercapnic HDT were statistically significant, unlike insignificant increases in TRT for the 70-day FARU study. However, increased peripapillary TRT was greater after 70 days when compared to 14 days of HDT bed rest at FARU^27,28^. We therefore speculate that in comparison to the FARU, more stringent HDT bed rest conditions such as greater posture control, increased time of bed rest, and other environmental factors (e.g., mild hypercapnia) could result in increased edematous changes at the level of the optic nerve head, rather than in a fundamental change (switch) to other (unknown) types of vascular and other physiological response.

As a consequence, we hypothesize that a retrospective study of the new strict HDT vascular images would demonstrate that increases in retinal vascular density exceeded those of the slight trends measured in the previous FARU study, although this currently remains unknown. Greater retinal edema with HDT than for the ISS may be associated with the trend toward increased vascular density reported here. Conversely, lesser retinal edema and greater choroidal edema after microgravity compared to HDT may be associated with the decreased vascular density measured by VESGEN for crew members. In a 21-day horizontal bed rest study without HDT, the diameters of the central retinal arteriolar and venular equivalents, another retinal vascular measure, decreased^36^. It is possible that as a terrestrial model of SANS, other postures of bed rest without HDT may be more consistent with our measures of retinal vascular decrease following microgravity, rather than with the trend toward retinal vascular increases from HDT bed rest, although many factors differ between our study and the 21-day study.

These differences in retinal vascular patterning and thickness following HDT and ISS missions may arise from somewhat different etiologic mechanisms that depend directly on the presence or almost complete absence of gravity. Other factors such as posture and the position of the head with respect to a terrestrial gravity vector are further discussed below. Edema is a generalized, nonspecific response to numerous pathologies throughout the body that compresses local tissues, and is present in many ocular disorders ranging from diabetic retinopathy to age-related macular degeneration and tissue injury^37–39^. Or the differences in retinal response to HDT and the ISS may perhaps depend on a different degree of exposure to elevated intracranial pressure as discussed elsewhere^5,9^. The variability in retinal vascular patterning and thickness measured by the two supine HDT studies discussed above^5,27^ suggests associations with other physiological factors such as body weight^6,40^, systemic cardiovascular status^2^, and perhaps genetics. Nutrition continues to be studied as potentially important for SANS in microgravity^41^ but has been standardized in these HDT studies.

While conditions for the ISS and HDT studies such as use of 30° Spectralis IR imaging reported here were comparable in many important ways, major differences include the length of exposure to the experimental environment (i.e., 70 days of HDT compared to six months on the ISS). The primary limitation of this proof-of-concept study is the low sample size, 16 astronaut retinas (8 subjects) and especially, 10 HDT retinas (5 subjects). Confidence in the results of decreased vessel density in astronauts was high due to low variability of the measured effect^25^, but low for increased vessel density with HDT because sampling was limited compared to variability in the effect. Further investigations are required to resolve more conclusively the apparent differences of retinal vascular response that would include larger cohorts, additional time points to define differences between six months on the ISS and the shorter durations of HDT and potentially, the more recent terrestrial spaceflight analog protocol in which the head and body are maintained in stricter HDT^42^. In addition, more comprehensive and conclusive associations between retinal vascular responses to HDT and the ISS and major ocular measures such as ODE, TRT, total choroidal thickness, globe flattening, visual acuity, and IOP would be important for such expanded studies that would test the accuracy of this proof-of-concept investigation.

Possible and nonexclusive pathophysiological mechanisms for decreased vessel diameter in microgravity include systemic losses in blood volume^43,44^ of approximately 10%, vascular compression due to optic disc/peripapillary swelling, and vessel obscuration resulting from subclinical edemas accompanied by tissue compression of the various ocular compartments. Venous pooling in the cephalad region, recently demonstrated for the internal jugular vein, may contribute to SANS and loss of blood volume in retinal vessels^7^. Conversely, the presence of constant gravity vector during 6° HDT may tend to pool both intravascular and extravascular fluids within the retina and ocular compartments, resulting in vessel dilation. New studies with higher resolution vascular ophthalmic imaging such as optical coherence tomography angiography (OCT-A) would resolve the questions raised by this study on differences of retinal vascular adaptations to microgravity and the HDT flight analog, although OCT-A is currently difficult to use on the ISS.

A further perspective is that the HDT findings suggestive of increased vascular density may indicate an initial response to the cephalad fluid shift on the ISS that during prolonged missions (i.e., greater than 70 days) accompanied by more strict exposure to microgravity may lead to a subsequent decrease in vascular density. As discussed above, further studies are needed not only to resolve these questions on the progression of SANS in microgravity, but also to determine whether vascular parameters at baseline, or a stress test such as a brief exposure to a greater degree of head tilt, can identify subjects at risk of developing SANS.

Overall, postural changes such as HDT and bed rest as spaceflight analogs have helped to advance our understanding of SANS. Both the similarities of ocular response such as optic disc edema and choroidal folds to the fluid shifts in postural changes and microgravity, as well as differences in choroidal and retinal thicknesses^5,27^ and in vascular patterning as reported for this study, are leading to a better understanding of the complex SANS etiology. However, the opposite vascular response in retinas to HDT and the ISS suggests that the presence and essentially complete absence of gravity in these two environments are of fundamental importance in determining the multiple physiological responses that are both similar and different. Nonetheless, the significance of the terrestrial gravity vector in HDT as a major factor cannot be so easily generalized. As a space flight analog, postural position and the angle of change from horizontal are major factors that modulate fluid shifts and physiological responses within the eye. On Earth, both supine and prone postures increase IOP compared to the upright and seated postures. For this first limited proof-of-concept study, the seated posture during pre- and post-HDT was chosen as most comparable to the seated postures of pre- and post-flight eye exams of astronauts. For the 70-day HDT study reported here, IOP increased by 1.79 mm Hg^27^. As reported and discussed in other studies, the downward-tilted prone position (HUT) may perhaps better mimic the ocular and associated cardiovascular changes during long-duration ISS missions than the supine (HDT) position^45–48^. Although changes in IOP during space flight appear variable and without overall increases in IOP 30 days prior to and 30 days after return from flight^2^, these previous results may be prone to measurement error. By a recent report, IOP normalizes to preflight values after 4 days of spaceflight^5^, and increased IOP has not been shown to be strongly associated with SANS. However, there is renewed interest in examining the role of IOP in space flight^9^. In a report of a mathematical model on the influence of supine posture and degree of HDT on IOP compared to horizontal posture, extension of the results on hydrostatic pressure and an autoregulatory component to the prone posture is discussed and commented that modeling the physiology of supine posture is more difficult^49,50^.

The VESGEN analysis, together with the SVPI, could be used to monitor and predict risks in crew vision and retinal vascular health for SANS during long duration missions, particularly for detection of early-stage ocular changes that are reversible by future countermeasures. The VESGEN v1.11 software freely and globally released by NASA in 2021^29^ contains a new segmentation option that automatically extracts binary vascular patterns by artificial intelligence (AI)/machine learning algorithms for faster, potentially more accurate vascular mapping and quantification. Nonetheless, larger studies beyond this proof-of-concept investigation are required to both confirm the results reported here and expand our understanding of the role of retinal vascular response to long-duration microgravity.

## Methods

### Participants

The HDT and ISS studies adhered to the tenets of the Declaration of Helsinki and following informed consent by participants, were approved by the Institutional Review Boards of The University of Texas Medical Branch, Galveston, Texas^27,28^, the NASA Johnson Space Center, Houston, Texas, and the NASA Lifetime Surveillance of Astronaut Health (LSAH)^25^. The 70-day HDT study (FARU) of healthy human subjects has been reported extensively elsewhere^27,28,51,52^. In brief, five healthy males and one female participated, and the average age was 39.5 ± 7.8 years. The cohort of eight healthy ISS crew members (16 retinas), for which the astronauts were recruited based on study data requirements for pre and post-flight OCT data, was described previously (7 males, 1 female; average age, 46.9 ± 5.4 years)^25^. Because there were no existing data on VESGEN measures of the eye surrounding HDT or spaceflight exposures, statistical power could not be estimated. Sample size was in part limited by costs and schedule. The primary aim of the study emphasized measuring and reporting pre to post-exposure changes over detecting statistically significant changes. The sample size of the HDT cohort (10 retinas, 5 subjects) was increased for the ISS cohort size of 16 retinas (8 crew members) to improve statistical precision for this group.

### Study design

As a comparison of previous HDT and ISS campaigns, the retrospective study was conducted in two phases. In the first phase of blinded vascular analysis, the HDT and ISS databases were established with de-identified retinal images that were masked as to pre-post status (i.e., databases were masked and randomized to remove subject identifiers and temporal sequence of acquisition). The ISS database comprised images of 16 retinas of eight crew members acquired shortly before and after six-month ISS missions (average mission time ± SD, 171 ± 17 days)^25^. The HDT database contained vascular images of the 12 retinas of six human subjects acquired approximately 12 days before and 2 days after 70 days of bed rest at 6° HDT. The retinas of one subject were omitted because the images were unanalyzable due to image blurring. The HDT sample was therefore limited to 10 eyes (5 subjects). Images were then analyzed with VESGEN as described below.

In the second phase, the vascular images and VESGEN results were unblinded and linked to (masked) subject identity and pre-post status. By statistical methods described below, vascular results were evaluated and compared to other ocular outcomes such as retinal thickness, IOP, and visual acuity.

### Imaging

Retinal images of the HDT and ISS cohorts were acquired by Spectralis^®^ infrared (IR) 30° imaging (Heidelberg Engineering GmbH, Heidelberg, Germany) as part of two separate research campaigns. Imaging conditions for the two studies were highly similar but differ in some respects. Details of the HDT and ISS imaging are compared in Supplementary Table 1, illustrated in Figs. 2–4, and described previously for both the ISS^25^ and HDT^27^ cohorts. For example, the Spectralis IR images were focused on the macular region in the HDT cohort and on the optic disc in the ISS cohort, as noted in Results. For accurate comparison of pre–post vascular differences, the Spectralis OCT AutoRescan™ feature was used in each cohort for the acquisition of follow-up images from the same retinal area as at baseline.

### Vascular analysis

The images were analyzed by trained vascular analysts in NASA’s VESGEN Laboratory according to the masked protocol described above. The binary vascular pattern of overlapping arterial and venous trees was first extracted from grayscale IR images by semi-automatic computer processing with Photoshop^®^ (Adobe, Mountain View, CA) using large, high-resolution Apple^®^ monitors (Cupertino, CA) as reported previously^18,19,24^. The extracted binary vascular pattern was separated into images of arterial and venous trees by comparison with color fundus photographs (when available) and by basic physiological principles of vascular tree connectivity, branching, vessel tapering, and arterio-venous pairing. Vessel interpretation was subject to agreement by at least two experienced vascular image analysts (with reviews and final decision by a senior analyst). The generational assignment of vessels into large (*G*_1-4_) and small (*G*_≥5_) groups was determined after early review of the arterio-venous maps and the associated quantified results for four or five retinas. Inspection of the vascular maps such as in Fig. 2 revealed that although subject to biological heterogeneity, the larger generations (*G*_1-4_) were generally characterized by relatively symmetric bifurcations that establish the basic arterial and venous arcades. As noted many times previously however, such as in a new review of VESGEN applications^24^, the most frequent branching events completing the retinal vascular arborizations are the offshoots of many smaller vessels from much larger vessels, although these numbers of offshoots are relatively limited in the low-resolution Spectralis IR vascular images. This generational group of smaller vessels, as measured for the study reported here, generally appears to remodel most actively^24,25^.

This is the first application of the VESGEN analysis to a longitudinal study of human retinal images acquired before and after defined time intervals (70 days and six months). For previous^19^ and concluding cross-sectional studies of diabetic retinopathy, only single images of patients were analyzed. Therefore a new method was developed for this study during the initial blinded vascular analysis phase for straightforward reconciliation of the pre/post binary vascular patterns and of the separated arterial and venous trees. The procedure was initiated to ensure that any differences detected by the vascular analysis could be attributed primarily to differences of vascular response to HDT and microgravity, and not to artifacts of the binary extraction and arterio-venous separation. In addition, although the quality of the pre/post images appeared highly uniform, minor differences in image detection of smaller vessels were expected to result from inevitable limitations such as contrast variation within the images. First, the retinal images were matched as pairs since each human retina displays a unique, individual branching pattern that is easily identifiable by visual inspection (Figs. 2–4). After the vascular binarizations were complete, the two binary images were compared to each other and to the grayscale images to resolve differences resulting from vessel interpretation, which were minor (estimated error, <5%)^18,19,25^. Remaining vessel differences were retained according to differences observed in the two grayscale images. Overall, the pre/post vascular patterns appeared highly similar by visual inspection for these healthy HDT and astronaut populations. Similarly, interpretations of the separated arterial and venous trees were compared and any differences, again minor, were resolved by inspection of the two grayscale images prior to unmasking of the pre/post status of the grayscale images.

Each binary arterial or venous image, together with the image calibration factor (Supplementary Table 1), served as the single input image for the automated mapping and quantification by VESGEN v1.05-1.10 (Fig. 2), written as a complex plug-in to ImageJ (U.S. National Institutes of Health)^53^ and now publicly available with user guide upon request to NASA^29^. Output includes arterial and venous maps, fractal dimension (*D*_f_), vessel tortuosity (*T*_v_), vessel diameter (*D*_v_), and densities of vessel number (*N*_v_), length (*L*_v_), area (*A*_v_) and branch point (*Br*_v_) for branching generations *G*_1_, *G*_2_,…*G*_x_^10–12,14,16,18,24,25,30^. The branching generations (*G*_1_–*G*_x_) are determined by relative decreases in vessel diameter and other considerations of vessel branching and tapering, based on the mechanics of laminar blood flow and also on experimental results for lung and heart vascular branching^19,24,54–56^. As examples, *L*_v1-4_ specifies *L*_v_ with respect to branching generations *G*_1_–*G*_4_ (Fig. 2) and physical dimensions such as *L*_v_ = 0.000775 μm/μm^2^ are notated as 7.75 × 10^–4^ μm/μm^2^.

### Statistics

Analyses were conducted using linear mixed-effects models that included subject-specific random effects (intercept and OD/OS) to account for repeated measures of the left (OS) and right (OD) eyes within individuals and across time. Primary VESGEN measures included *D*_f_, *L*_v_, *L*_v1-4_, and *L*_v≥5_ for each vascular tree (arteries or veins), and each measure was modeled independently. Robust sandwich standard errors (heteroskedastic-consistent) were used to account for the heterogeneity of variance between groups and across time. Categorical fixed effects were included for time (pre or post) and group (HDT or crew) as an interaction allowing for differential response between groups. An overall F-test determined significance for at least one time and group combination being different from the others. Pairwise comparisons were conducted when the overall F-test was statistically significant. Specifically, pairwise comparisons were conducted on within-group pre to post change (post - pre), as well as the contrast of those changes between groups. Testing for these comparisons was conducted via *t*-tests on the expected marginal means. Analysis of pre- to post-HDT change in subjects is reported in Table 2, and contrasted to space-flight related change in crew in Table 1.

Similarly, mixed models were used to assess the association of VESGEN measures and ocular parameters (RNFL thickness, peripapillary retinal thickness, macular retinal thickness, IOP, spherical equivalent refraction, and near and distance visual acuity logMAR) in the HDT subjects. For this analysis, the models included fixed effects for time and each ocular measure as a covariate (in separate models). Coefficients, standard errors, *p*-values, and corresponding false discovery rates are reported in Table 3.

Models were fit using the GLIMMIX procedure in SAS v9.4. The LSMEANS and LSMESTIMATE statements were used to estimate marginal means and conduct pairwise comparisons (pre to post change within each group, and contrasting those changes between groups). Estimates of the expected marginal means for each time and group combination and comparison included the means, standard errors, confidence limits, and *p*-values.

Residual plots were visually inspected to ensure appropriate conformity to normality assumptions. The VESGEN measures did not deviate from normality and no transformation was necessary. Due to sample size limitations, no adjustments were made to account for multiple testing across the VESGEN measures. The number of tests conducted compared to the limited samples limits interpretation to hypothesis generating, i.e., determining potential areas for confirmatory study.

### Software availability

The VESGEN software with extensive User Guide is publicly available upon request to NASA (https://software.nasa.gov/software/ARC-17621-1).

### Reporting summary

Further information on research design is available in the Nature Research Reporting Summary linked to this article.

## Supplementary information

Supplementary Information

Reporting Summary

## Data Availability

VESGEN vascular results along with associated clinical parameters for the FARU HDT and ISS crew member populations are available in NASA’s Life Sciences Data Archive (LSDA https://lsda.jsc.nasa.gov/Request/dataRequestFAQ) upon request.
