# Supplementary material for: Opposite response of blood vessels in the retina to 6° head-down tilt and long-duration microgravity
Source: NPJ Microgravity. 2021 Oct 14;7:38. doi: 10.1038/s41526-021-00165-5 (PMC8516890; doi:10.1038/s41526-021-00165-5)
Supplement: Supplementary file 1 — Supplementary Information [file 41526_2021_165_MOESM1_ESM.pdf]

## Supplementary Information

Supplementary Table 1 Scanning Conditions by 30° Heidelberg Spectralis IR for HDT Bed Rest and ISS Crew Members

| IR Scan Conditions | HDT Bed Rest* | ISS Crew Members* |
|---|---|---|
| Posture | Standard upright position | Standard upright position |
| Centered on: | Macula (fovea) | Optic disc |
| Resolution | Low<br>768×768 pixels<br>≈11.4 µm/px | High [2x greater]<br>1536×1536 pixels<br>≈5.8 µm/px |
| Post-imaging processing | Contrast sharpening | Heidelberg images were transformed by NASA LSAH to bmp images of lower image resolution (496×496 pixels) prior to VESGEN analysis. Images therefore were rescaled for VESGEN study back to original physical dimensions by dividing with scaling factor adjustment (0.3229)[25]. |
| Pre and post imaging | Before, approximately 12 days<br>After, within 2 days | Before, 144 days ± 100 days<br>After, 4 days ± 1.7 days |
| Time between image acquisitions | 70 Days (approximate) | 6 Months (approximate) |

Scanning conditions and instruments varied somewhat between the two studies, resulting in different capture efficiencies of smaller vessels. Very minor differences in imaging resolution were generated by Heidelberg software for each retinal scan. ≈, approximately equal to or asymptotically equal to.
*Imaging conditions summarized here for HDT and crew members documented previously[25, 27]

Supplementary Table 2 Fractal Dimension ($D_f$) Before and After Head Down Tilt (HDT)

| Subject | HDT Status | Right, Arterial | Right, Venous | Left, Arterial | Left, Venous |
|---|---|---|---|---|---|
| 1 | Pre<br>Post | 1.30<br>1.30 | 1.34<br>1.32 | 1.38<br>1.37 | 1.33<br>1.33 |
| 2 | Pre<br>Post | 1.33<br>1.35 | 1.33<br>1.35 | 1.30<br>1.37 | 1.36<br>1.36 |
| 3 | Pre<br>Post | 1.36<br>1.41 | 1.37<br>1.37 | 1.36<br>1.35 | 1.38<br>1.42 |
| 4 | Pre<br>Post | 1.36<br>1.37 | 1.32<br>1.34 | 1.35<br>1.35 | 1.34<br>1.35 |
| 5 | Pre<br>Post | 1.28<br>1.33 | 1.30<br>1.35 | 1.31<br>1.30 | 1.31<br>1.36 |

Cutoff for $D_f$ in skeletonized images as vascular increase or decrease, $\Delta D_f > |0.02|$ (dark gray, vascular increase; hatch marks, vascular decrease). Transitional cases, $\Delta D_f = |0.02|$ (light gray).
Time points for retinal imaging for 70 days of bed rest are -11 or -12 days before and +2 days after.
Best-fit linear correlation for $D_f$ by box-counting method in all images: $r^2 \geq 0.97$.
